# Experiments on Vapor Pressure of KCl at Different Molar Fractions in Mixtures of KCl + K_2_SO_4_

**DOI:** 10.1021/acsomega.2c03896

**Published:** 2022-10-09

**Authors:** Xiuhua Li, Fang He, Frank Behrendt, Alba Dieguez-Alonso, Haiquan Sun, Hailiang Wang

**Affiliations:** † Shandong Vocational College of Science and Technology, Weifang, Shandong261053, China; ‡ Shandong University of Technology, Zibo, Shandong255049, China; § Technische Universität Berlin, Berlin10623, Germany; ∥ Otto-von-Guericke-Universität Magdeburg, Magdeburg39106, Germany; ⊥ Weifang Institute of Technology, Qingzhou, Shandong262500, China

## Abstract

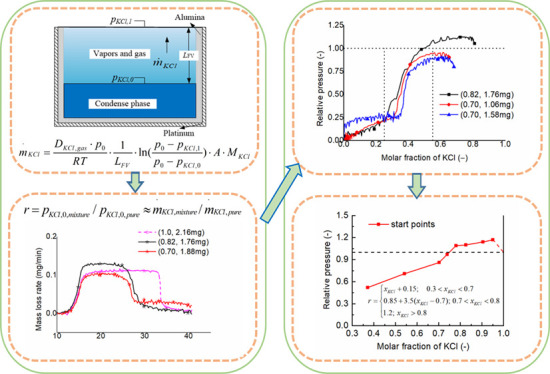

The objective of this paper is to measure the vapor pressure of potassium chloride (KCl) in its mixture with potassium sulfate (K_2_SO_4_). Evaporation behavior of pure salts of KCl, K_2_SO_4_, and their mixtures at different molar fractions were examined using a simultaneous thermogravimetric analyzer (STA) at different temperatures with a pair of crucibles (outer: platinum; inner: alumina). The dependence of the vapor pressure of KCl on its molar fraction in mixtures of KCl + K_2_SO_4_ was obtained on the basis of relative pressure. Results show that vapor pressure of KCl is increased to 1.2 times when a small amount of K_2_SO_4_ (molar fraction from 0.05 to 0.20) is added. Evaporation of KCl will be inhibited by K_2_SO_4_ when the molar fraction of K_2_SO_4_ is higher than 0.27, which is its fraction in the eutectic system of KCl + K_2_SO_4_. Vapor pressure of KCl decreases significantly with increasing molar fractions of K_2_SO_4_ at its inhibition scope.

## 1 Introduction

Inorganics escaping from the solid matrix to the gas phase during thermal conversion of solid fuels,^1,2^ incineration of solid wastes,^3^ and metallurgical treatment of iron ores^4,5^ often lead to fouling of heat exchangers, particulate emission, and corrosion of refractories. Evaporation is one of the main routes of this inorganics release, especially for alkali compounds.^2,6−9^ Quantification of the evaporation rate, in which the saturated vapor pressure of the salt is the most important data, plays a great role in determination of the optimal operation condition.

Vapor pressure (*P*_s_) of pure salt is often calculated according to the Antoine equation (log *P*_s_ = −52.23*B*/*T* + *C*, where *P*_s_ is in millimeter of mercury; *T* is temperature in kelvin; *B* and *C* are constants, and the related constants are often calculated or measured).^10,11^ In many industrial systems, mixtures of salts instead of pure salts are often present. For example,^12,13^ potassium chloride (KCl) and potassium sulfate (K_2_SO_4_) are the main components in the solid phase during combustion of agro stalks,^14^ with many other salts and eutectic melting often occurring. In the mixture of molten salts, the vapor pressure of each salt often differs significantly from that of pure salt. For an ideal mixture, it is described according to Raoult’s law. For a real molten mixture, reliable data on vapor pressure of each salt can only be obtained by experiments and the database is far from complete.^15,16^ Lack of data makes it difficult to predict the evaporation rate of salts in real industrial systems. Many methods have been developed to measure this vapor pressure,^15−17^ some are quite complex and time consuming. According to our previous studies and pre-experiments, the mass loss rate and heat effect of pure salts and mixtures can be determined in a simultaneous thermogravimetric analyzer (STA). Vapor pressure of salt in mixtures might be obtained via well-designed experiments.

The objective of this paper is to examine the vapor pressure of KCl in its mixture with K_2_SO_4_ at different molar fractions and different temperatures using an STA.

## 2 Materials and Methods

### 2.1 Materials

Pure KCl (>99.5%) and K_2_SO_4_ (>99.0%) were acquired from Tianjin Fengchuan Chemical Reagent Co. Ltd. and then crushed into powders with a particle size of <120 mesh using an agate mortar. The powders were dried at 105 °C for 24 h in an electric oven with an air blower. After cooling, eight types of mixtures of KCl + K_2_SO_4_ with molar fractions (KCl versus K_2_SO_4_) of 0.37, 0.54, 0.70, 0.73, 0.78, 0.82, 0.90, and 0.95 were prepared. Powders of pure salts and mixtures were sealed in plastic bags and stored in a desiccator for the experiments.

### 2.2 Measurements of the Evaporation Process

**Figure 1:**
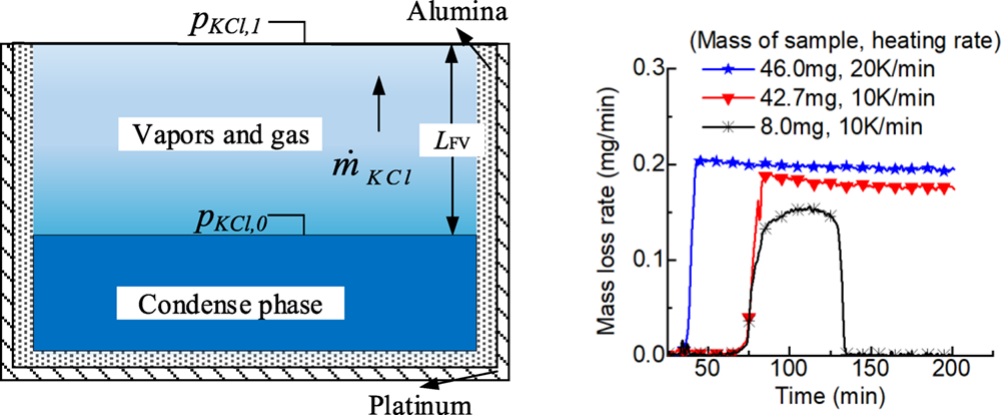
Evaporation of KCl from crucibles in an STA (left) and the characteristics of evaporation rates at different sample sizes (right, data from ref (7)).

Mass loss and heat effect of pure salts and their mixtures were examined using an STA (TGA/DSC 1/1600, weighing accuracy: 0.01%, enthalpy accuracy: ±10%). Potassium salts are reactive at high temperature and could probably damage the instrument. Two measures were taken to avoid corrosion of the instrument by the sample he as shown in Figure 1.

The first is to use a pair of crucibles (inner: 50 μL of alumina, outer: 70 μL of platinum) in all runs. Normally, alkali salts could leak from an alumina crucible to pollute/damage the sensor model of the instrument and could injure a platinum crucible at high temperature. By using a pair of crucibles, the outer platinum crucible can prevent direct contact of a little leaked salts with the sensor model of the instrument. The inner alumina crucible can reduce the direct contact of the sample with the platinum crucible and consequently prevent the damage. In expanding of experiments to solid waste, such as biomass ash, it can also prevent the sintering of samples on a platinum crucible, which is much more expensive than an alumina crucible.

The second is to use only a small amount of the samples. According to our previous experiments^7^ shown in the right side of Figure 1 and many pre-experiments, small size samples can also get a stable evaporation rate, although it is not as good as big samples (the detailed analysis is discussed later).

After each blank run, a sample of approx. 1.6 mg of KCl was put into the inner crucible and heated from 100 °C to the set temperatures (875, 900, 925, and 950 °C) at a heating rate of 50 °C/min. Nitrogen with a flow rate of 150 mL/min was used as purge gas and nitrogen with a flow rate of 20 mL/min was used as protect gas. It should be noted that the maximum temperatures of the samples in each run are 871, 896, 921, and 946 °C, although the temperatures of the references are 875, 900, 925, and 950 °C, which are the same as the set temperatures.

### 2.3 Quantification of Vapor Pressure of KCl in Mixtures on the Basis of Relative Pressure

Pre-experiments showed that evaporation of K_2_SO_4_ below 950 °C is negligible and the mass loss rate in each run of mixtures of KCl and K_2_SO_4_ is the evaporation rate of KCl. Vapor pressure of KCl on the top surface of the condense phase dominates this mass loss rate of KCl from molten salts as shown in Figure 1. The evaporation process is the same with Stephen flow and the mass loss rate (*m*·_KCl_, kg/s) is given in eq 1:^7,18^
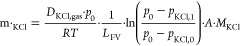
1where *A* denotes the surface area of the condense phase (m^2^); *D*_KCl, gas_ denotes the diffusion coefficient of KCl in gas (m^2^/s) ();^7,19^*L*_FV_ denotes the height of the free volume in a crucible (m); *M*_KCl_ denotes the molar mass of KCl (kg/mol); *p*_0_, *p*_KCl,0_, and *p*_KCl,1_, denote pressure of the atmosphere, vapor pressure of KCl at the top surface of the condense phase and pressure of KCl at the outer surface of a crucible (Pa); *R* denotes the universal gas constant (J/(mol K)); *T* denotes temperature of the sample (K).

Since the flow rate (150 mL/min) of purge gas above the crucible is high, it is assumed that *p*_KCl,1_ at the outer surface of the crucible equals 0. Equation 1 transforms into eq 2.
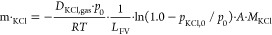
2

After runs of pure KCl and a mixture of KCl + K_2_SO_4_ in an STA, mass loss rates are obtained. If *T* and *L*_FV_ in eq 2 are well controlled, relationship of the mass loss rate in the mixture and in pure KCl with vapor pressure of KCl in the mixture and pure KCl is given in eq 3.
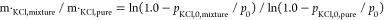
3where *m*·_KCl, mixture_ and *m*·_KCl, pure_ denote evaporation rates of KCl in mixtures and in pure KCl (kg/s); *p*_KCl,0, mixture_ and *p*_KCl,0, pure_ denote vapor pressures of KCl in mixtures and in pure KCl (Pa). When *p*_KCl,0, mixture_/*p*_0_ ≪ 1.0, which is the case in evaporation of KCl in most combustion and incineration processes, eq 3 can be simplified into eq 4.
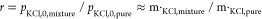
4where *r* is the relative pressure, which is the ratio of vapor pressure of KCl in mixtures to that of pure KCl. Since vapor pressure of pure KCl can be accurately calculated using Antoine’s equation (*P*_s_ = 133 × 10^–52.23*B*/*T*+*C*^),^7^ vapor pressure of KCl in the mixture can be quantified using this relationship of relative pressure.

It should be noted that detailed information of variables and constants involved in eqs 1–4 has been reported in references (7) and (14).

## 3 Results and Discussion

### 3.1 Reproducibility of Experiments and Effects of the Heating Rate/Sample Size

**Figure 2:**
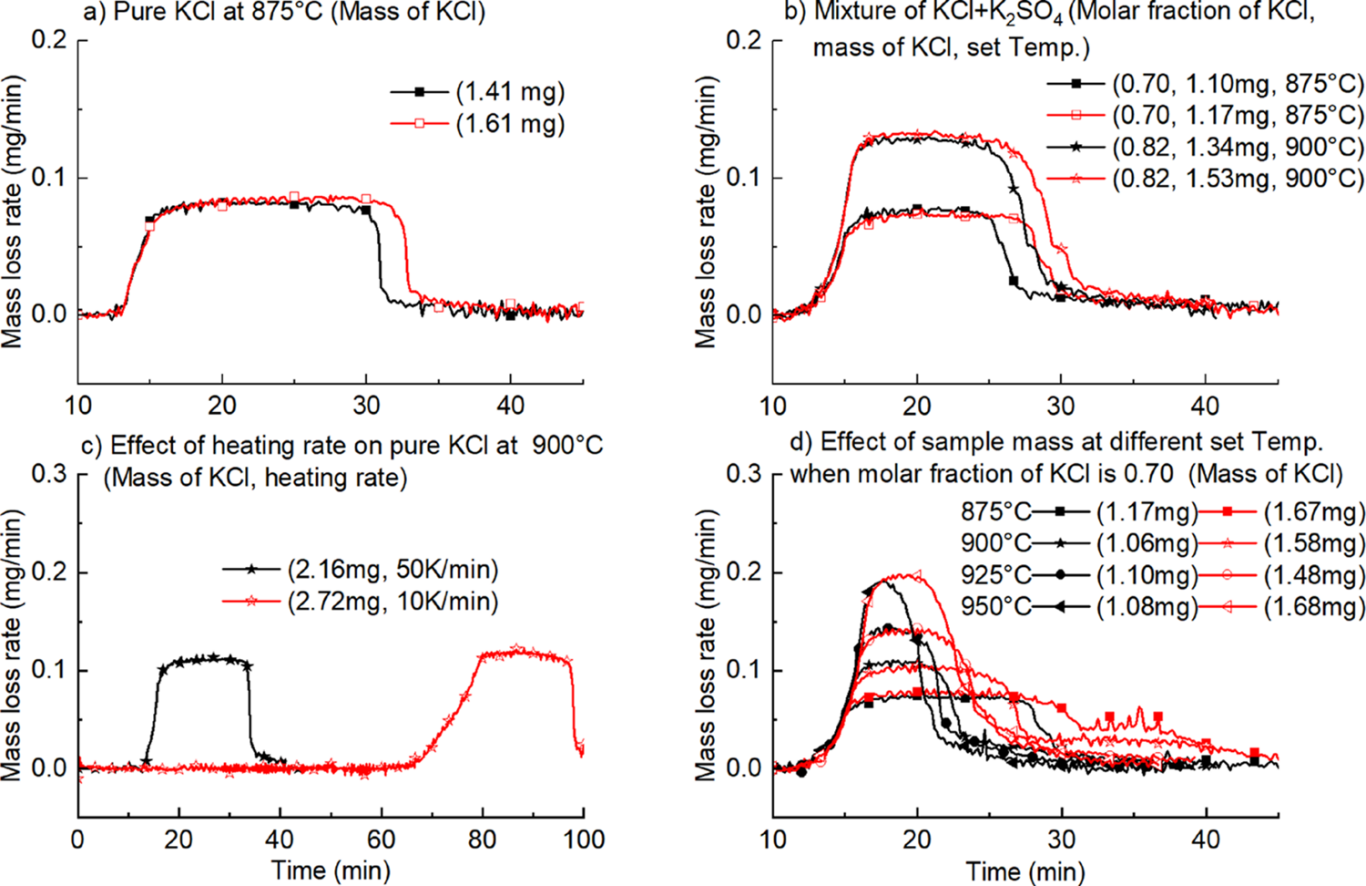
Reproducibility of experiments and effects of experimental conditions.

Mass loss rates from the experiment are the key parameter in quantifying the vapor pressure as shown in eq 4. In order to check the reproducibility of experiments and the sensitivity of operation conditions, several runs were repeated and runs at different heating rates and different masses of the sample were made. Results of mass loss rates with data on the molar fraction of KCl, mass of KCl, set temperature, and heating rate in the respective legends are shown in Figure 2.

The mass loss rate in all runs remains almost constant in most part of the set temperature and drops at the last stage of evaporation. Reproducibility of the mass loss rate at the constant stage in repeated experiments is high with a relative error of less than 5% as shown in subfigures (a) and (b).

The effect of the heating rate from 10 to 50 °C/min on the evaporation rate at the constant stage is shown in subfigure (c). The mass loss rate increases slightly for about 3 min after the arrival of set temperature at a heating rate of 50 °C/min. More evaporation occurs before set temperature at a heating rate of 10 °C/min than that of 50 °C/min, but the evaporation rate is almost not affected. In addition, the vapor pressure of potassium chloride (KCl) in its mixture is obtained on the basis of relative pressure, which is calculated on the basis of the ratio of the mass loss rate of KCl in mixtures to that of pure KCl according to eq 4. Therefore, a high heating rate has little effect in our experiments, and it was adopted for most runs due to much less time (40 min vs 110 min) required.

The effect of increasing the sample mass of KCl from 1.0 to 1.7 mg on the evaporation rate at most constant stages is shown in subfigures (a), (b), and (d) and it is also negligible. It is reasonable that more sample mass has a longer stage of constant mass loss rates. The long stage of constant evaporation rate shows that the process is not sensible to *L*_FV_ in certain scope of the sample mass. As a consequence, eq 4 can be applied in determining the vapor pressure.

### 3.2 Evaporation Behavior of Pure Salts and Mixtures

**Figure 3:**
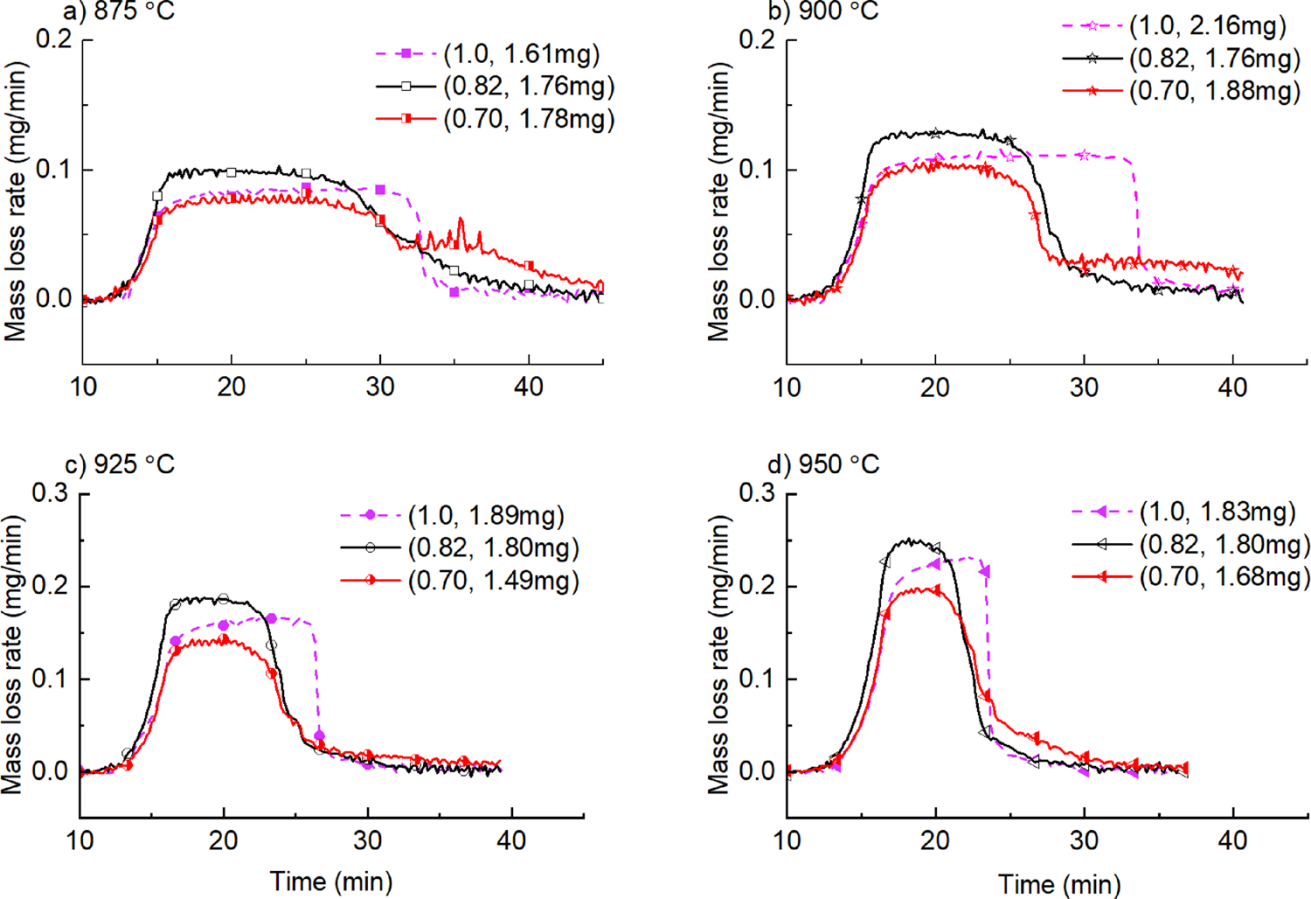
Evaporation behavior of pure salts and mixtures (legends show the molar fraction of KCl and mass of KCl in the original sample).

Mass loss rates of KCl from pure KCl and mixtures of KCl + K_2_SO_4_ at molar fractions of 0.82 and 0.70 (mass ratios of KCl to K_2_SO_4_ are 2:1 and 1:1) at different temperatures are shown in Figure 3. Two characteristics can be seen from all the subfigures. The first is that the molar fraction of KCl in the mixture affects the evaporation rate significantly. The evaporation rate of a mixture with a molar fraction of 0.82 is significantly higher while that of 0.70 is significantly lower than that of pure KCl. According to Roult’s law in which partial pressure is assumed to be in linear correlation with its molar fraction (always <1.0), the evaporation of KCl in different molar fraction mixtures should be inhibited by K_2_SO_4_ via decreasing vapor pressure of KCl.^14^ However, the fact is that vapor pressure of KCl in a mixture is higher than that in pure KCl under some conditions, and this is far away from Roult’s law.

The second observation is that the evaporation rates drop more slowly in all mixtures than that in pure KCl at the last stage of evaporation. More K_2_SO_4_ (molar fraction of 0.70 compared to that of 0.82) leads to a longer stage of low evaporation rate. This implies that the existence of K_2_SO_4_ affects the vapor pressure of KCl at the last stage (lower molar fraction of KCl due to its evaporation) significantly.

### 3.3 Vapor Pressure of KCl in the Mixture of KCl + K_2_SO_4_

**Figure 4:**
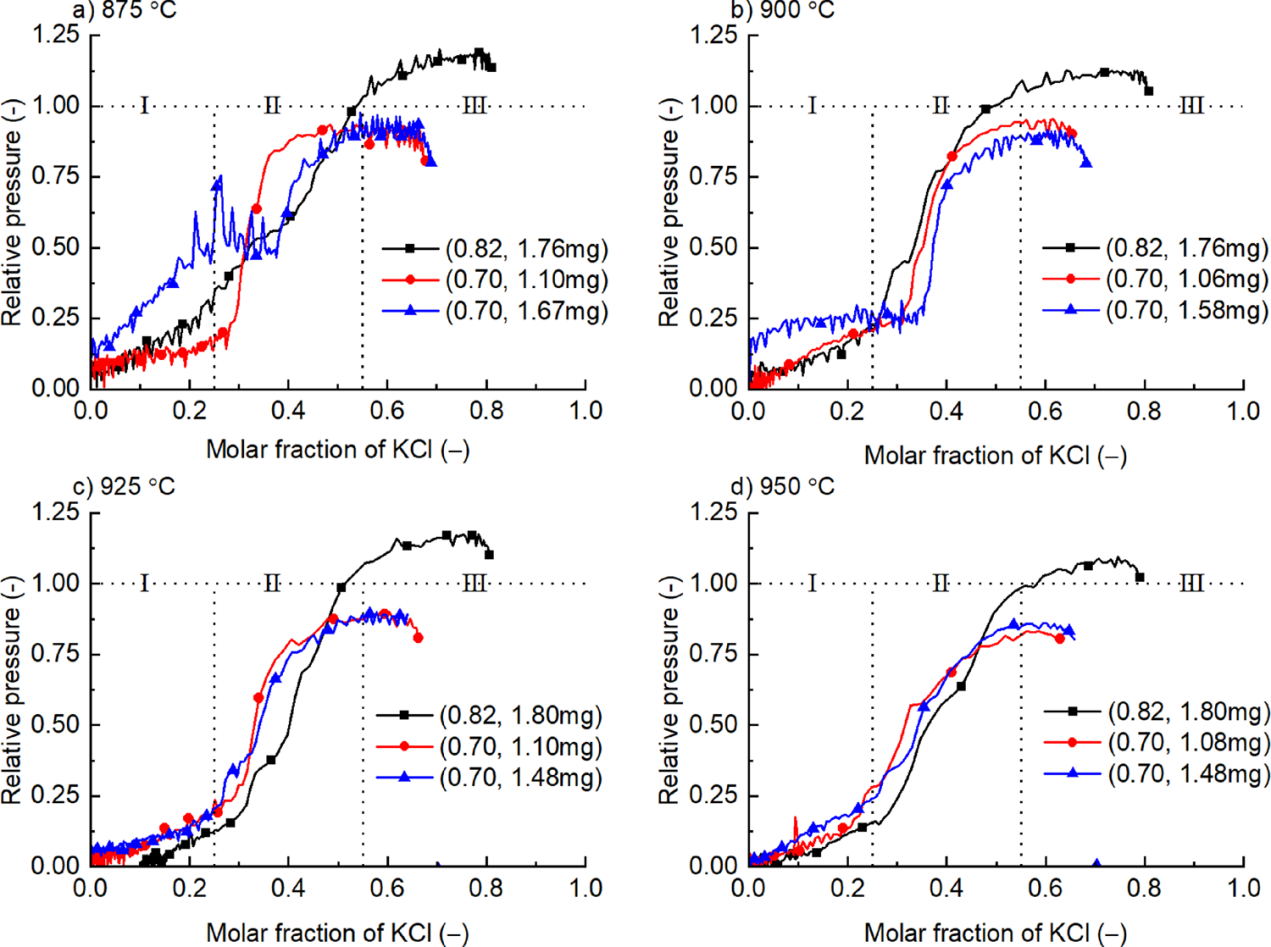
Relative vapor pressure of KCl in the mixture with its molar fraction at different temperatures (legends show the molar fraction of KCl and mass of KCl in the original sample).

In order to identify the effect of the molar fraction of KCl on its vapor pressure in the mixture, eq 4 was used in processing of all the data from Figure 3 with the results shown in Figure 4.

X-axis is the molar fraction of KCl. During the process (from right to left in each curve), it decreases with the evaporation of KCl and is calculated as eq 5.
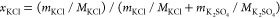
5where *m*_KCl_ denotes the mass of KCl remained in the sample (kg). *m*_K_2_SO_4__ denotes the mass of K_2_SO_4_ in the original sample (kg). *M*_KCl_ and *M*_K_2_SO_4__ denote the molar mass of KCl and K_2_SO_4_ (kg/mol).

*Y*-axis is the relative pressure *r*, which is the ratio of vapor pressure of KCl in the mixture to that in pure KCl and is calculated using eq 4.

**Figure 5:**
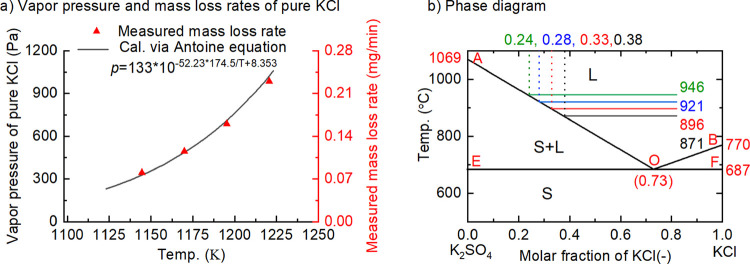
Relationship of the calculated vapor pressure of pure KCl with measured mass loss rates (a) and the phase diagram of KCl and K_2_SO_4_ (b).

In eq 4, *m*·_KCl, pure_ was chosen 3 min after the maximum temperature has been arrived in the runs of pure KCl. They are 0.086, 0.115, 0.16, and 0.23 at a sample temperature of 871, 896, 921, and 946 °C, respectively. The ratios between these values agree well with ratios of vapor pressure of pure KCl calculated from Antoine’s equation as shown in Figure 5a. This indicates that the measurement is reliable.

Characteristics of relative pressure at different temperatures share similarities as shown in Figure 4. There are three stages (*I*, *II*, and *III*) in all curves—slow change at a low molar fraction (<0.25), significant change at a medium molar fraction (0.25∼0.55), and a stable stage at a high molar fraction (>0.55) of KCl.

In stage I, the relative pressure increases slowly with the increasing fraction of KCl. Higher temperature leads to less deviation among experiments. This is partly due to the high signal/noise ratios in measurement of the mass loss rate at high temperatures. Another reason is the effect of solid in the mixtures. Figure 5b shows the binary phase melting diagram of KCl-K_2_SO_4_. In Figure 5b, four groups are classified—the mixture of liquid KCl and K_2_SO_4_ (area above the AOB line), the mixture of liquid KCl-K_2_SO_4_ and solid K_2_SO_4_ (area on the left of the AOE line), the mixture of liquid KCl-K_2_SO_4_ and solid KCl (area on the right of the BOF line), the mixture of solid KCl and K_2_SO_4_ (area under the EF line). At sample temperatures of 871, 896, 921, and 946 °C, there is a solid phase in the mixture of KCl + K_2_SO_4_ when the molar fraction of KCl is very low. It is seen that solid K_2_SO_4_ appears when the molar fraction of KCl is less than 0.38 at a temperature of 871 °C. When the molar fraction is lower than 0.24, the solid phase appears in all the above temperatures. If there is solid in the molten mixture, molar fraction calculation using eq 5 cannot represent the state of solution and it will also affect the flow of vapors. In combustion of biomass, some of low-melting eutectic mixtures (such as KCl/NaCl with other components) often appear and salting-out effects on molar fraction calculation using eq 5 and vapor flowing are similar.

In stage II of Figure 4, the relative pressure of KCl increases significantly with an increasing molar fraction and the maximum relative pressure is normally <1.0. It implies that K_2_SO_4_ inhibits the evaporation of KCl at this stage. In stage III, relative pressure is almost constant. The value is affected by the original condition of each run, especially the molar fraction of KCl in the original sample. The relative pressure from the mixture with molar fractions of 0.70 and 0.82 is 0.85 and 1.2, respectively.

### 3.4 Turning Point from Promotion to Inhibition of Evaporation of KCl by K_2_SO_4_

**Figure 6:**
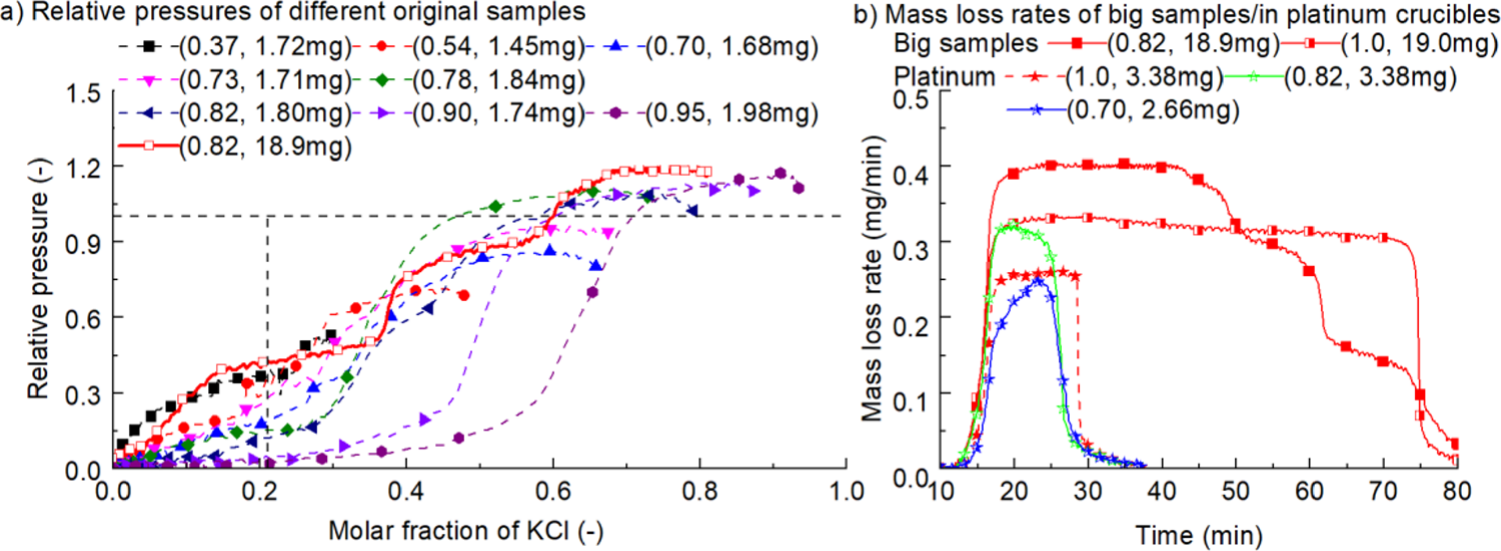
Effect of initial conditions on the relative pressure of KCl and the effects of sample size, the kind of crucible on the mass loss rate (legends show the molar fraction of KCl and mass of KCl in the original sample).

Further experiments on evaporation of KCl at 950 °C in an STA from different original samples with the molar fraction of KCl from 0.35 to 0.95 have been performed and the results are shown in Figure 6. Results of all runs show that the evaporation is significantly promoted when the molar fraction of KCl is higher than 0.78. The promotion changes only slightly with the molar fraction of KCl in the scope of 0.78 to 0.95. This implies that adding only a little K_2_SO_4_ (its molar fraction from 0.05 to 0.22) into KCl will promote evaporation of KCl significantly. A similar phenomenon was recorded that CsI enhances the evaporation of the cation elements composing FLiNaK.^15^ The mechanism of promoting evaporation is left for future work.

Figure 6a shows that evaporation of KCl is inhibited when the molar fraction of KCl is lower than 0.73, in which the molar fraction of K_2_SO_4_ is higher than 0.27. According to Figure 5b, in a eutectic system of KCl + K_2_SO_4_, the molar fraction of KCl is also 0.73. It means the turning point from promotion to inhibition of evaporation of KCl by K_2_SO_4_ is the same with its fraction in the eutectic system. Supplement repeated experiments and experiments at other temperatures (875, 900, and 925 °C) show the same results. It can be concluded that the evaporation of KCl is inhibited by K_2_SO_4_ in most combustion conditions because molar fractions of K_2_SO_4_ under these conditions are normally higher than that in the eutectic system.

### 3.5 Effect of the Original Sample and the Reason

**Figure 7:**
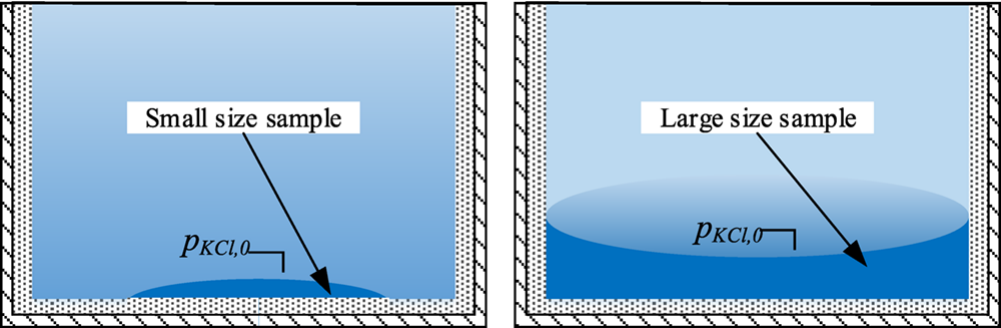
Outer surface of the molten sample at different sample sizes.

Theoretically, for the slow process of evaporation, the relative pressure should be the same at the same molar fraction of KCl in the mixture, regardless of the initial condition. Thus, curves in the middle stage of Figure 6a should overlap. However, there is a significant difference if the original sample mass differs. The reason might be the small sizes of the sample which will not cover the whole area of the bottom of the crucible as shown in Figure 7. The significant differences in the amount of K_2_SO_4_ among different original samples will also affect the evaporation rate.

Supplement experiments with big samples (0.82, 18.9 mg; 1.0, 19.0 mg) and samples in a platinum crucible have also been performed. The mass loss rates are shown in Figure 6b. For big samples, the evaporation rate of pure KCl is reasonable. However, when the mass drops from 19.0 to 1.83 mg, the evaporation rate is 0.32 mg/min, much larger than 0.23 mg/min observed in experiments with an original mass of 1.83 mg as shown in Figure 3d. This indicates that the evaporation rate is affected by the original mass. This might be explained by the coffee-ring phenomenon^20^ which states that the contact line of a liquid drop is pinned to its original outer surface. The original surface of the sample varies with its mass and the larger sample has a larger outer surface as shown in Figure 7.

Another remarkable effect is that the history of the evaporation rate is a staircase curve for the mixture. Observation shows that K_2_SO_4_ climbs quickly out of the inner alumina crucible and sticks to the outer wall of the platinum crucible during the heating of the mixture, even samples that are less than half full in the crucible are loaded. The staircase curve might be related to this.

For the samples in a platinum crucible, characteristics of the evaporation rate are similar to those in crucibles (outer: platinum; inner: alumina) shown in Figure 3. Climbing of K_2_SO_4_ also occurs when only a platinum crucible is used. The fleeing of K_2_SO_4_ from the condense phase changes the molar fraction of KCl in the mixture. All this shows that there is interaction between salts and some crucibles.

Anyway, the relationship of relative pressure of KCl with its molar fraction is calculated according to eq 4 on the basis of data from supplement experiments of large size and the result is also shown in Figure 6a. It is seen that the promotion and inhibition function are present and the scaling up factor in the promotion stage is also 1.2. Of course, data in stage II is not accurate due to the interaction of the crucible. For samples in a platinum crucible, scaling factors of 1.26 and 0.85 were also obtained at the initial stage when molar fractions of KCl in the mixture are 0.82 and 0.70. It indicates that the evaporation at the initial stage does represent vapor pressure even when there is interaction between the sample and the crucible.

If a type of crucible which has ignorable interaction with salt is found, using large-size samples is recommended to speed up the measurement. By comparing the evaporation rate of the mixture and the pure salt, the vapor pressure at different molar fractions at certain temperature would be obtained.

### 3.6 Relationship of Vapor Pressure with the Molar Fraction of KCl

**Figure 8:**
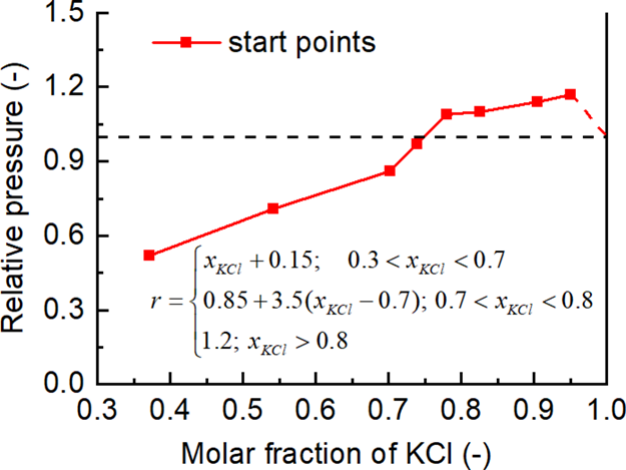
Relative pressure of KCl in mixtures of KCl and K_2_SO_4_.

In order to get reliable information from the runs performed, only the starting point of each run is taken into account and the relationship of relative pressure with the molar fraction of KCl is shown in Figure 8. The turning point from promotion to inhibition is seen from the figure. It is also seen that the inhibition effect increases significantly with the decreasing molar fraction of KCl at the inhibition scope. A rough correlation for theoretical analysis of the industrial process is obtained via piecewise linearization and it is also shown in Figure 8.

The result can be used as a reference in analyzing the emission of KCl in combustion of solids and the investigation can also be used as a reference in the measurement of similar salts. For agro stalks, KCl accounts for less than 50% of weight in soluble fractions of ash prepared from low temperature combustion^13^ and the evaporation of KCl during combustion is normally inhibited. The measurement method is now limited to vaporization of one salt. For vaporization of several salts simultaneously, the mathematical model is also necessary, and this is left for future work.

## 4 Conclusions

Measurement of the vapor pressure of salt in its mixture with other salts is quite complex. In this study, vapor pressure of KCl in the mixture of KCl + K_2_SO_4_ at different molar fractions and different temperatures were analyzed on the basis of relative pressure using an STA. It shows that small amounts of K_2_SO_4_ added to the mixture increases the vapor pressure of KCl to 1.2 times. The molar fraction of K_2_SO_4_ at the turning point from promotion to inhibition of evaporation of KCl approximates 0.27, which is its fraction in the eutectic system of KCl and K_2_SO_4_. Vapor pressure of KCl decreases significantly with increasing molar fractions of K_2_SO_4_ at the inhibition scope. A rough correlation of vapor pressure with molar fraction is determined and the result can be used as a reference in analyzing the emission of KCl in combustion of solids.
